# B-Lymphoblastic Lymphoma Isolated to the Testes: A Case Report and Review of Literature

**DOI:** 10.7759/cureus.51284

**Published:** 2023-12-29

**Authors:** Aimee Daccache, Edwin Feghali, Krystel Chedid, Kahlil Saad, Jack Staddon

**Affiliations:** 1 Pediatrics, University of Kansas School of Medicine-Wichita, Wichita, USA; 2 Internal Medicine, Lebanese American University School of Medicine, Beirut, LBN; 3 Urology, University of Kansas School of Medicine-Wichita, Wichita, USA; 4 Pediatric Hematology/Oncology, University of Kansas School of Medicine-Wichita, Wichita, USA

**Keywords:** b lymphoblastic lymphoma, chemotherapy, pediatric oncology, testicular mass, testicular lymphoma

## Abstract

Isolated testicular involvement in pediatric lymphoma is rare and poses diagnostic challenges. In this study, the case of an isolated testicular B-lymphoblastic lymphoma in a 9-year-old boy is discussed with an emphasis on the difficulties in diagnosing and treating such an unusual presentation. This example illustrates the importance of considering lymphoblastic lymphoma in the differential diagnosis of an unidentified source of testicular enlargement. Furthermore, it highlights the possible efficacy of systemic chemotherapy with or without surgical excision. The article advances our knowledge of this unusual clinical situation.

## Introduction

Lymphoblastic lymphoma comprises approximately 25% of pediatric non-Hodgkin lymphoma (NHL), of which only 20-30% is B-lymphoblastic lymphoma (B-LLy), while the remaining 70-80% of cases are T-lymphoblastic lymphoma (T-LLy) [1]. Given that only about 800 cases of pediatric NHL are diagnosed in the United States each year, B-LLy is quite rare, with an estimated 40-60 cases of B-LLy expected to occur annually in the United States [1]. B-LLy is distinguished from the morphologically identical and far more common B-lymphoblastic leukemia by the presence of less than 25% lymphoblasts in the bone marrow, regardless of nodal or extra-nodal involvement [2]. 

In contrast to T-LLy which has a predilection to the mediastinum (thymic/mediastinal mass), B-LLy is more commonly found in nodal and extranodal sites [2]. For example, in a well-characterized European cohort of 53 pediatric B-LLy patients, the sites of primary disease were mostly in bone, skin or subcutaneous tissue, bone marrow, and lymph nodes [3]. Less common sites included the chest, gonads, central nervous system, gastrointestinal tract, and kidneys. Only 4 patients (7.5%) had gonad primaries, including 2 testicular primaries (7.4% of 27 male patients), only one of whom had isolated testicular disease. Building on this foundational knowledge, we present an unusual case of isolated primary testicular B-LLy in a 9-year-old boy. 

## Case presentation

A 9-year-old boy presented to the emergency room with a 2-day history of abdominal pain. He was previously healthy with no pertinent medical history. He had no fever, night sweats, or weight loss but did have a recent mildly decreased appetite. He had no vomiting, diarrhea, constipation, cough, upper respiratory symptoms, fatigue, muscle aches, mouth lesions, nosebleeds, or blood in stools. He had no family history of cancer. 

Right testicular swelling was identified the following day by his primary care provider, who referred him immediately for scrotal ultrasound which ruled out torsion but did show a 2 cm mass. He was then referred to urology. Urologist exam demonstrated warm and erythematous right hemiscrotum and moderately swollen and very tender right testicle. A 2-week course of trimethoprim-sulfamethoxazole was prescribed for epididymo-orchitis with abscess. Follow-up ultrasound showed an enlarged right testicle measuring 4.2 x 2.2 x 2.9 cm, with increased vascularity, and epididymis with hyperemia again suggestive of right epididymo-orchitis. A complex appearing hypoechoic collection in the right testicle measured 2.2 x 1.5 x 1.9 cm without significant internal vascularity, concerning for intratesticular abscess. He was treated with additional courses of oral antibiotics. After more than 2 months from presentation, he continued to have some testicular discomfort and a still enlarged right testicle. He then underwent a right inguinal orchiectomy. 

**Figure 1:**
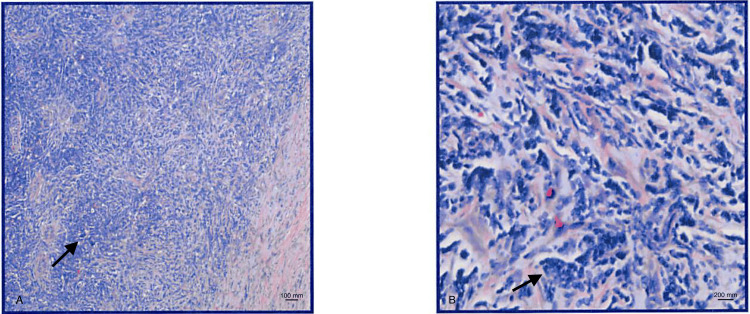
Testicular biopsy demonstrating small blue cell proliferation, morphologically compatible with lymphocytes/lymphoblasts. (A) Magnification 40x; scale bar 100 mm; (B) Magnification 200x; scale bar 200 mm.

Morphologic and immunostain findings supported a diagnosis of B-lymphoblastic leukemia/lymphoma. Pathology showed a small blue cell proliferation suggestive of lymphocytes or lymphoblasts (Figure 1).

Tumor marker testing prior to orchiectomy was negative for increased alpha-fetoprotein and beta-human chorionic gonadotropin. 

He was admitted for port-a-cath placement, bone marrow evaluation, lumbar puncture, and acute lymphoblastic leukemia/lymphoma induction chemotherapy. Bone marrow aspirate and biopsy were negative including by flow cytometry at a detection level of 0.01%. Bone marrow cellularity was normal at 80%. CSF was also negative for blasts by both morphology and flow cytometry. A whole-body PET-CT scan done after the orchiectomy showed slight nodular uptake in the right inguinal region thought most likely to be a benign post-operative finding, without evidence of regional lymph node involvement nor disseminated disease. Although in surgical remission, he was started on intensive chemotherapy per Children’s Oncology Group protocol AALL1732 to prevent recurrence. Fifteen months after diagnosis, in his maintenance phase of treatment, he remains in remission. 

## Discussion

Testicular involvement in pediatric acute lymphoblastic leukemia may occur at diagnosis or relapse but is unusual as an isolated finding outside of the relapsed setting [4]. Primary testicular lymphoblastic lymphoma is considered if the disease is restricted to the testicle after excluding bone marrow involvement at diagnosis. To the best of our knowledge, only seven cases of primary B-LLy have been previously reported in the literature [3,5-9], only five of which were completely isolated to the testicle (Table 1).

**Table 1:** Reported primary testicular B-lymphoblastic lymphoma. *Presented at age 9, diagnosed at 10 years of age. ALL, Acute lymphoblastic leukemia; CHOP, cyclophosphamide, doxorubicin (hydroxydaunorubicin), vincristine (Oncovin), prednisone; DFCI, Dana Farber Cancer Institute; Hyper-CVAD, Hyper-fractionated cyclophosphamide, vincristine, doxorubicin (Adriamycin), and dexamethasone; NED, No evidence of disease.

ISOLATED TESTICULAR LYMPHOMA AT DIAGNOSIS				
Article	Age (Years)	Symptoms	Treatment	Outcomes
Ducassou et al. [3]	Child (age not reported)	No data	Chemotherapy: Systemic & CNS directed therapy per LMT 96, or EORTC 58881/58951. Local control: Details not reported.	NED (duration not provided).
Garcia et al. [5]	3	Left testicle pain and enlargement	Surgery: Biopsy only. Chemotherapy: Pediatric ALL protocol DFCI 05-001. Radiation: None.	NED (24 months).
Daccache et al. [this report]	9*	Right testicle swelling, abdominal pain, decreased appetite	Surgery: Total orchiectomy. Chemotherapy: Intensive systemic and intrathecal chemotherapy per AALL1732. Radiation: None.	NED (14 months).
Binesh et al. [6]	13	Left testicle swelling, moderate tenderness	Surgery: Total orchiectomy. Chemotherapy: High dose systemic and intrathecal chemotherapy; intensive consolidation (Protocol M). Radiation: None.	NED (8 months).
Sahni and Desai [7]	19	Left testicle swelling	Surgery: High left inguinal orchiectomy. Chemotherapy: Systemic (CHOP); CNS prophylaxis chemotherapy. Radiation: Contralateral testis. Relapse treatment: CHOP	Relapse to left breast (3 months after primary treatment). Alive (40 months).
Tombolini et al. [8]	39	Left testicle acute pain, palpable hard mass in inferior left testicle	Surgery: Orchifunicolectomy. Chemotherapy: 6 cycles of hyper-CVAD followed by methotrexate and cytarabine. Radiation: Contralateral testis.	NED (6 months).
TESTICULAR PRIMARY TUMOR WITH METASTATIC DISEASE AT DIAGNOSIS				
Article	Age (Years)	Symptoms/Other sites of disease	Treatment	Outcomes
Ducassou et al. [3]	Child (age not reported)	Symptoms not stated. Bone marrow involved at diagnosis.	Chemotherapy: Systemic & CNS-directed therapy per LMT 96. Local control: Details not reported.	Bone marrow relapse after 1.2 months.
Zhu et al. [9]	27	Bilateral testicular swelling, mild pain. Bone marrow: 2.5% involvement by flow cytometry at diagnosis.	Surgery: Core needle biopsy only. Chemotherapy: Hyper-CVAD protocol alternating with high-dose methotrexate and cytarabine. Radiation: None.	Bone marrow leukemia relapse (8 months, ~2 months after treatment); suicide while on relapse therapy.

We report another rare case of isolated testicular B-LLy to further expand our knowledge and understanding of this rare presentation. 

Differentiating malignant from inflammatory testicular processes can be challenging. Scrotal enlargement and pain, the presenting symptoms in our patient, are most frequently secondary to vascular compromise or inflammation. The most common causes include torsion of the testicular appendages, epididymitis, torsion/detorsion of the spermatic cord, and testicular trauma [10]. Scrotal ultrasound is the initial modality of choice in evaluating isolated testicular swelling and can help distinguish intra-testicular from extra-testicular abnormalities [11]. In lymphomas, ultrasound typically shows diffuse or focal areas of hypoechogenicity with normal ovoid shape as well as hypervascularity [11]. However, no specific findings on ultrasound can reliably differentiate lymphoma from other testicular tumors, and a biopsy may be necessary. In our patient’s case, symptoms persisted despite adequate treatment with antibiotics, so the decision for orchiectomy was made and pathology showed B-LLy infiltration. 

Our patient presented with local symptoms only (abdominal and testicular pain and testicular swelling), and similar to the other cases reviewed (Table 1) did not have significant systemic symptoms. Staging evaluations of our patient showed no evidence of disease elsewhere, which some might consider an indication of Stage 1 disease [6]. Nevertheless, as the International Pediatric Non-Hodgkin Lymphoma Staging System now defines ovarian involvement as Stage III [12], we chose, by analogy with ovarian involvement, to classify our patient’s testicular involvement as Stage III as well. As a Stage III lymphoma, we considered his testicular B-LLy to be high risk and chose to treat him with intensified systemic chemotherapy per AALL1732 [13]. 

Testicular lymphomas of other histologic subtypes (mostly diffuse large B-cell lymphoma) in adults have a high risk of extranodal relapse, even with a history of localized disease at diagnosis [14]. Data regarding primary testicular B-LLy in children and adults, largely limited to anecdotal data from case reports (Table 1), suggest that aggressive multimodal therapy can often be successful, at least when B-LLy is limited to the testes without bone marrow involvement at diagnosis. Whether intensive induction chemotherapy can safely eliminate the need for local control with surgery and/or radiation, as is often the case for testicular involvement in childhood acute lymphoblastic leukemia, is unknown [4]. 

## Conclusions

Testicular presentation of B-lymphoblastic lymphoma is very rare but should be considered in the differential of patients with isolated testicular swelling of unknown origin. If this diagnosis is made, treatment with systemic acute lymphoblastic leukemia/lymphoma chemotherapy appears promising. Further research is needed to optimize therapeutic strategies and enhance our understanding of this rare condition. 
